# The Potential Use of DCE-MRI Texture Analysis to Predict HER2 2+ Status

**DOI:** 10.3389/fonc.2019.00242

**Published:** 2019-04-12

**Authors:** Zejun Jiang, Lirong Song, Hecheng Lu, Jiandong Yin

**Affiliations:** ^1^Shengjing Hospital of China Medical University, Shenyang, China; ^2^School of Sino-Dutch Biomedical and Information Engineering, Northeastern University, Shenyang, China

**Keywords:** HER2, DCE-MRI, texture analysis, machine learning, breast cancer

## Abstract

**Purpose:** To evaluate the ability of texture analysis of breast dynamic contrast enhancement-magnetic resonance (DCE-MR) images in differentiating human epidermal growth factor receptor 2 (HER2) 2+ status of breast tumors.

**Methods:** A total of 73 cases were retrospectively selected. HER2 2+ status was confirmed by fluorescence *in situ* hybridization. For each case, 279 textural features were derived. A student's *t*-test or Mann-Whitney U test was used to select features with statistically significant differences between HER2 2+ positive and negative groups. A principal component analysis was applied to eliminate feature correlation. Three machine learning classifiers, logistic regression (LR), quadratic discriminant analysis (QDA), and a support vector machine (SVM), were trained and tested using a leave-one-out cross-validation method. The area under a receiver operating characteristic curve (AUC) was measured to assess the classifier's performance.

**Results:** The AUCs for the different classifiers were satisfactory, ranging from 0.808 to 0.865. The classification methods derived with LR and SVM demonstrated similarly high performances, and the accuracy levels were 81.06 and 81.18%, respectively. The AUC for the classifier derived with SVM was the highest (0.865), and a marked specificity (88.90%) was presented. For the classifier with LR, the AUC was 0.851, and the corresponding sensitivity (94.44%) was the highest.

**Conclusion:** The texture analysis for breast DCE-MRI proposed in this study demonstrated potential utility in HER2 2+ status discrimination.

## Background

Human epidermal growth factor receptor 2 (HER2) is an orphan tyrosine kinase receptor. It is reported that HER2 is overexpressed in 15–20% of breast cancers. Overexpression of HER2 in breast cancer correlates with shortened disease-free survival. Compared with HER2-negative patients, patients with HER2-positive breast cancers have a poor prognosis, a high probability of lymph node metastasis and a high risk of recurrence (1–3). However, previous studies have demonstrated that patients with HER2-positive breast cancers have a favorable clinical response to trastuzumab (4, 5). It is therefore critical to determine the HER2 status of a patient for treatment selection as well as for predicting therapeutic response. Two methods have been adopted in the field for detecting HER2 status: immunohistochemistry (IHC) and fluorescence in situ hybridization (FISH). HER2 expression is typically divided into four categories: 0, 1+, 2+, 3+. For HER2 IHC measurements, a score of 0 or 1+ are considered negative, and a score of 3+ is considered positive. For HER2 2+ cases, IHC cannot be used to confirm this status, and HER2 2+ status must instead be tested using FISH (6, 7). However, the FISH assay is typically cost-prohibitive. In addition, further testing is time-consuming and often can delay treatment. A novel cost-effective and rapid method to identify HER2 2+ status is urgently needed.

Dynamic contrast enhancement-magnetic resonance imaging (DCE-MRI) is the most sensitive modality for the detection of breast cancer at present (8–12). A study by Kuhl et al. indicates that contrast enhancement with MRI itself represents an imaging biomarker (12). Additionally, DCE-MRI allows for the assessment of tumor heterogeneity, which can be quantified by textural features (13). Previous studies have demonstrated that texture analysis can be effectively applied to distinguish molecular subtypes of benign and malignant breast lesions and invasive breast cancer (14–16). Moreover, Sardanelli et al. demonstrated that HER2 amplification is associated with angiogenesis, which can be measured by DCE-MRI (17, 18). Therefore, we presume that the texture analysis of breast DCE-MRI scans can be utilized to successfully discriminate between HER2 2+ positive and negative status.

To our knowledge, there have not been any previous reports demonstrating HER2 2+ categorization based on texture analysis of breast DCE-MRI scans. In the current study, we propose and evaluate this novel supplementary tool for distinguishing between HER2 2+ positive and negative breast cancers.

## Materials and Methods

### Patient Cohort

**Figure 1:**
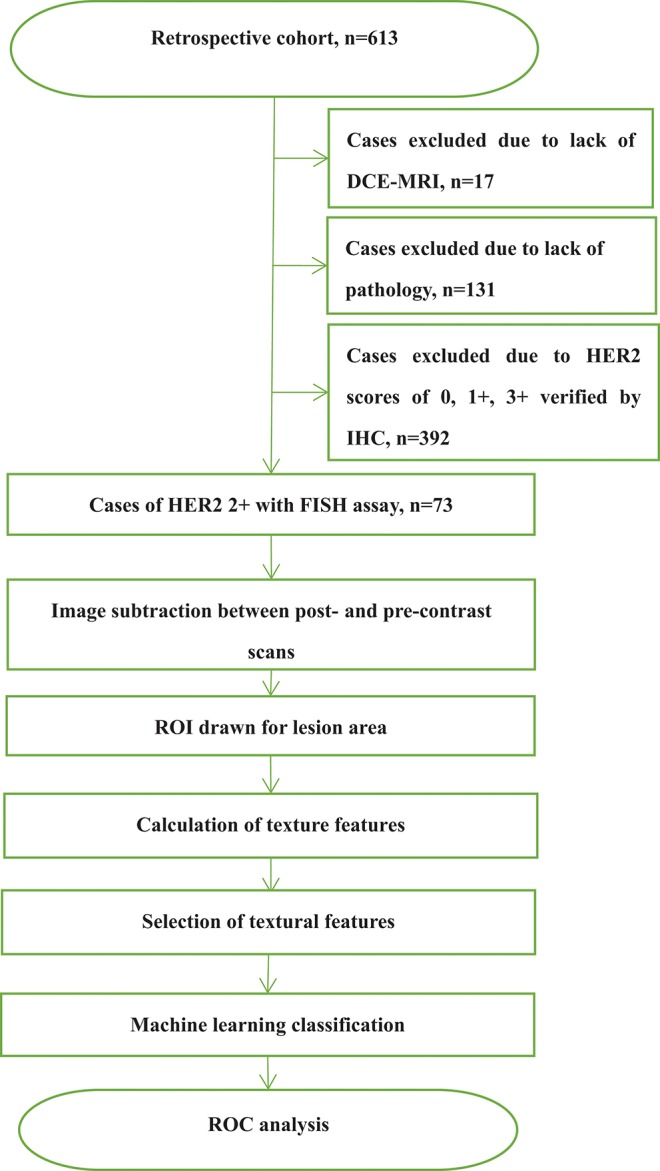
Flowchart of the methods for determination of HER2 2+ status based on texture analysis.

This study was approved by the Shengjing Hospital Institutional Ethical Committee. As this was a retrospective study, written informed consents from patients were waived. Figure 1 outlines a flowchart of the methods used for HER2 2+ discrimination. In total, 73 patients were enrolled in the study. All patients received DCE-MRIs and had a breast carcinoma confirmed by pathology or biopsy. For the included cases, 37 (50.68%) were HER2 2+ positive and 36 (49.32%) were HER2 2+ negative. HER2 2+ status was finally verified using FISH, which is considered as the gold standard in the field. In fact only **two** types of cancers, invasive ductal carcinomas (IDCS) and ductal carcinoma *in situ* (DCIS), met the case inclusion criteria. The details of patients selected for subsequent analyses are listed in Table 1.

**Table 1:** Details of selected cases with HER2 2+ status confirmed by FISH.

**Characteristic**		**FISH status**		**Number**
		**Positive**	**Negative**	
**AGE (YEAR)**				
	20–29	1	0	1
	30–39	8	8	16
	40–49	8	14	22
	50–59	16	6	22
	60–69	4	8	12
**MEAN DIAMETER (MM)**				
		22.23	19.36	
**MRI BI-RADS**^**θ**^				
	3	1	0	1
	4A	0	1	1
	4B	6	2	8
	4C	15	25	40
	5	12	6	18
	6	3	2	5
DCE-MRI TIC*				
	Inflow	2	1	3
	Plateau	6	11	17
	Washout	29	24	53
**PATHOLOGY**				
	DCIS	0	2	2
	IDCS	37	34	71
**IHC**				
	ER positive	17	32	49
	ER negative	20	4	24
	PR positive	24	28	52
	PR negative	13	8	21
	Ki-67 ^#^≥ 14%	29	24	53
	Ki-67 ^#^ < 14%	8	12	20

θ *Breast imaging reporting and data system*.

* *Time Intensity Curve*.

# *When Ki-67 frequency was >14%, the status of Ki-67 staining was considered positive (19)*.

### Image Acquisition

DCE-MRI was performed with a GE 3.0 T MRI scanner (Signa HDxt, GE Healthcare, USA). All patients in our study were scanned in the prone position using a dedicated eight-channel double-breast coil. The orientation of slice image was transverse. During each MRI examination, a pre-contrast series of VIBRANT-VX sequence T1-weighted 3D images were initially captured. Eight post-contrast scans were acquired after the intravenous injection of a contrast agent (0.5 mmol/mL, Gadodiamide, Omniscan, GE Healthcare, USA; Magnevist, Bayer-Shering Pharmaceuticals) at 4 mL/s (0.15 mmol/kg bodyweight). An equal volume of saline flush was used at the same flow speed. The imaging parameters were as follows: repetition time (TR) 7.42 ms, echo time (TE) 4.25 ms, flip angle 15°,slice thickness 2.20 mm, spacing between slice 2.20 mm, inversion time 20 ms, image matrix 1,024 × 1,024, temporal acquisition 80 s, slice number 78. Prior to texture analysis, a slice image with the maximum size of the lesion was selected from the subtracted volume.

Next, many texture features would be measured with a freely available software, and those with statically significant difference were selected. In addition, in order to eliminate the correlation among the significantly different features, a principal component analysis (PCA) was applied.

### Texture Analysis

Image texture analysis was carried out using the professional software, MaZda (version 4.6.0, Institute of Electronics, Technical University of Lodz, Lodz, Poland), which can be publicly accessed (20, 21). A region of interest (ROI) covering the lesion area was manually drawn by an experienced breast radiologist. The image intensities inside the ROI were normalized between μ ± 3σ (μ, mean of image intensity; σ, standard deviation). The range was then quantified to 8 bits/pixel. This method allows for balancing of the brightness and contrast variations and minimization of the variability introduced from inter-scanner differences (22). A total of 279 texture features were derived from the histogram, co-occurrence matrix, run-length matrix, absolute gradient, autoregressive model, and wavelet (Table 2). A detailed background of these textural features can be found in pertinent published literature (23, 24).

**Table 2:** The features calculated with MaZda using different texture analysis methods.

**Methods**	**Texture features**	**Number**
Histogram	Mean, variance, skewness, kurtosis, 1% percentile, 10%percentile, 50% percentile, 90% percentile, 99% percentile	9
Co-occurrence matrix*	Angular second moment (ASM), contrast (CON), correlation (COR), sum of squares (SOS),inverse difference moment (IDM), sum average (SA), sum variance (SV), sum entropy (SE),entropy (ENT), difference variance (DF),difference entropy (DE)	220
Run-length matrix^#^	Run length non-uniformity (RLN), gray level non-uniformity (GLN), long run emphasis (LRE), short run emphasis (SRE), fraction of image in runs (FIR)	20
Absolute gradient	Mean, variance, skewness, kurtosis, percentage of pixels with non-zero gradient	5
Autoregressivemodel	Teta1, teta2, teta3, teta4, sigma	5
Wavelet	Wavelet parameters	20
Total		279

* *Co-occurrence matrix-based parameters were computed for four directions (0, 90, 45, and 135°) and the distance is represented by values of 1, 2, 3, 4, and 5. (d, 0), (0, d), (d, d), and (d, –d) represent 0, 90, 45, and 135°, respectively, where d is the distance. For example, S(0,1)ASM represents a distance of 1 and direction of 90°*.

# *The run-length matrix-based parameters were computed for four directions (0, 90, 45, and 135°)*.

### Statistical Analysis

Some features measured above were not beneficial for HER2 2+ categorization, and instead increased the complexity of subsequent machine learning. Therefore, statistical analyses performed with SPSS (version 19.0, Chicago, IL, USA) were carried out to reduce the number of weak features. The features with statistically significant differences between HER2 2+ positive and negative groups were selected for subsequent analyses. A Kolmogorov-Smirnov test for each kind of feature was first performed to test whether the samples had a normal distribution (25). If the distribution was normal (*P* ≥ 0.05), a Student's *t*-test was used to investigate the differences between the HER2 2+ positive and negative groups (25). Otherwise, the median value for the Mann-Whitney *U* test was used (25). Furthermore, to eliminate the correlation among the significantly different features, a principal component analysis (PCA) was applied (26). MATLAB 2018a (Mathworks, Natick, MA, USA) was used for the classifier application. Three popular and efficient machine learning methods [logistic regression (LR), quadratic discriminant analysis (QDA) (27), and a support vector machine (SVM)] were used for the classifier, respectively. To avoid over-fitting, the leave-one-out cross validation (LOOCV) was used to assess classification performance (28). In LOOCV, one sample was used as the test dataset while the remaining samples were utilized as the training set.

To assess the performance of the classifiers in determining HER2 2+ status, a receiver operating characteristic (ROC) curve for each method was drawn using the professional statistics software, MedCalc (version 14.10.20, http://www.medcalc.org/). The area under the ROC curve (AUC) provided automatically was used as an index of diagnostic performance. The specificity and sensitivity were also measured, which were used to calculate the accuracy.

## Results

**Figure 2:**
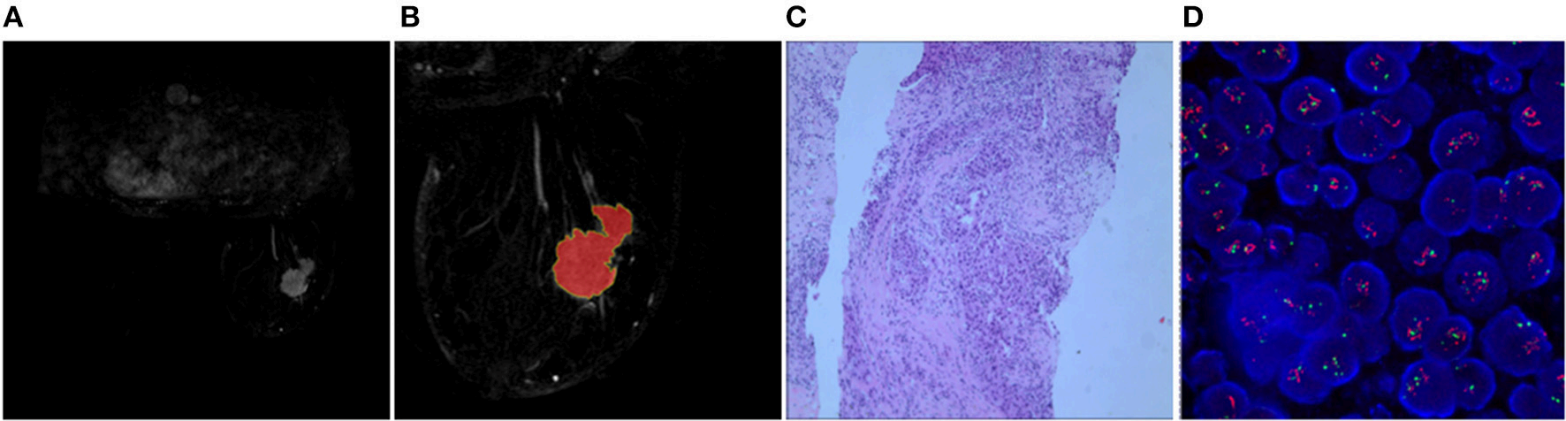
Results obtained from a randomly-selected HER2 2+ positive case. **(A)** Subtraction image of pre- and post-contrast scans (regular mass and BI-RADS 5). **(B)** Enlarged image showing the ROI (red region) delineated manually by an experienced radiologist. **(C)** Pathology results showing IDCS (HER2 2+ gene confirmed by IHC). **(D)** Positive HER2 subtype tested by FISH (HER2/CEP17 > 2.2).

**Figure 3:**
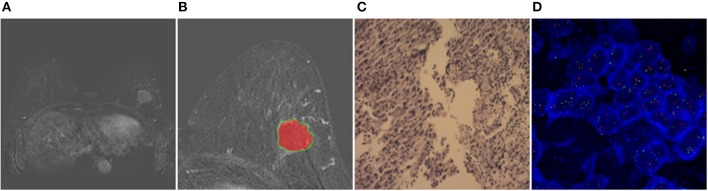
Results obtained from a randomly-selected HER2 2+ positive case. **(A)** Subtraction image of pre- and post-contrast scans (regular mass and BI-RADS 4C). **(B)** Enlarged image showing the ROI (red region) delineated manually by an experienced radiologist. **(C)** Pathology results showing IDCS (HER2 2+ gene confirmed by IHC). **(D)** Negative HER2 subtype tested by FISH (HER2/CEP17 < 2.2).

**Figure 4:**
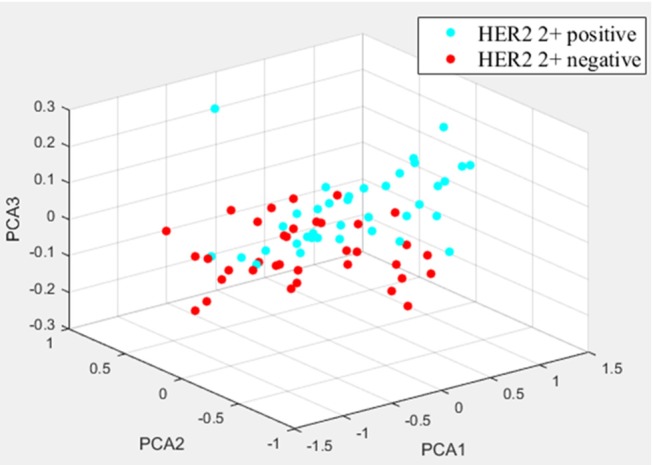
The distribution differences of three types of PCA-derived components of HER2 2+ positive and negative groups.

**Figure 5:**
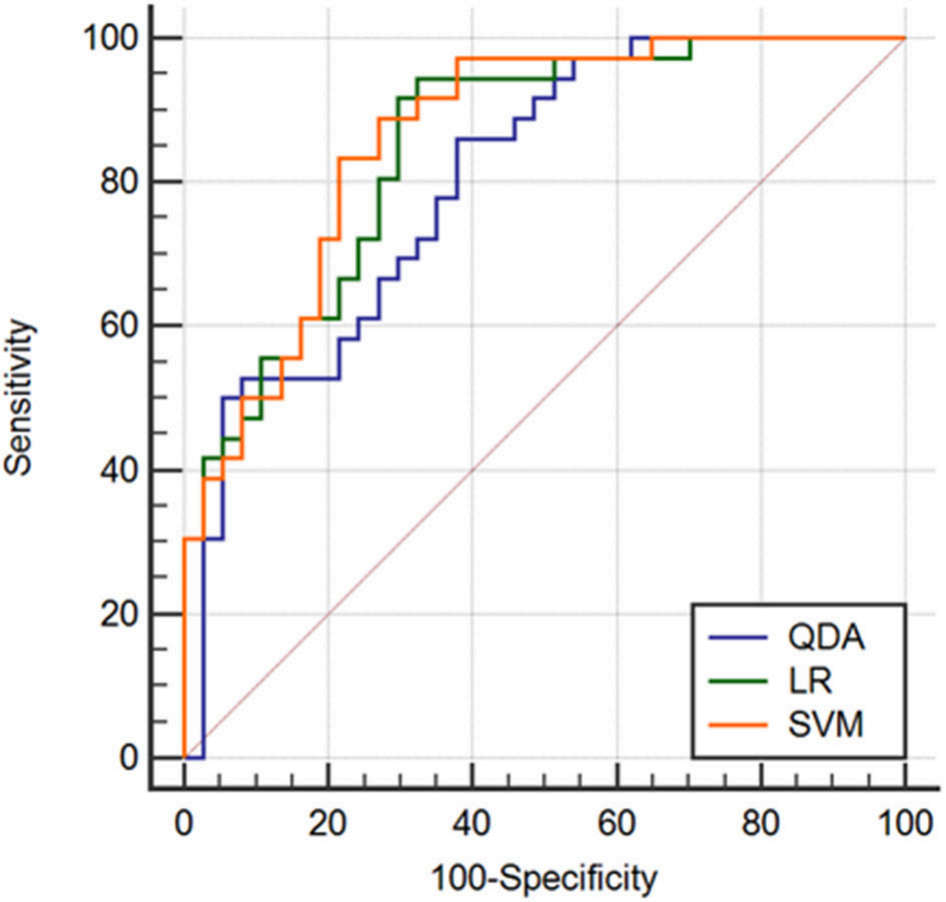
Comparison of ROC curves derived from different machine learning methods.

Two randomly-selected cases are shown in Figures 2, 3, where the subtraction images, lesion ROI, pathology and FISH results are presented in sequence. The features with statistically significant differences are listed in Table 3. Figure 4 shows a scatter plot created by the three types of components derived from the PCA. The distribution difference between HER2 2+ positive and negative groups is demonstrated in this figure. The ROC curves for the performance evaluation are shown in Figure 5. The corresponding AUCs, specificity, sensitivity as well as accuracy are listed in Table 4. All the AUC values are >0.80, which demonstrated the potential value of our proposed method for determining HER2 2+ status. Among the classifying methods, LR and SVM performed similarly. Classifiers using SVM achieved the highest AUC (0.865) and had a marked improvement in specificity. Classifiers using LR were relatively more accurate (81.18%) and specific (94.44%). Overall, the LR and SVM classifiers performed better than the QDA classifier.

**Table 3:** Features with statistically significant differences as measured by a Student's *t*-test or Mann-Whitney *U*-test.

**Parameters**	**HER-2 2+** **positive**		**HER-2 2+** **negative**		***P*-value**
	Mean ± SD*	**Median**	Mean ± SD*	**Median**	
S(1,0)ASM		0.002		0.001	0.043
S(1,0)IDM		0.917		0.914	0.018
S(1,0)DE	1.379 ± 0.126		1.284 ± 0.130		0.020
S(0,1)ASM		0.002		0.001	0.045
S(0,1)SE		2.001		2.043	0.050
S(0,1)DE		1.313		1.357	0.007
S(1,1)IDM		0.071		0.064	0.003
S(1,1)ENT	2.900 ± 0.377		2.739 ± 0.297		0.049
S(1,1DE	1.492 ± 0.141		1.398 ± 0.151		0.008
S(1,-1)DE	1.495 ± 0.141		1.397 ± 0.144		0.005
S(2,0)DE	1.583 ± 0.136		1.485 ± 0.150		0.005
S(0,2)IDM		0.054		0.050	0.013
S(2,2)IDM		0.046		0.041	0.039
S(2,2)DE	1.644 ± 0.165		1.550 ± 0.166		0.019
S(2,-2)DE	1.644 ± 0.168		1.539 ± 0.174		0.012
S(3,0)DE		1.552		1.613	0.013
S(0,3)DE	1.649 ± 0.164		1.552 ± 0.169		0.016
S(3,3)IDM	0.037 ± 0.012		0.043 ± 0.011		0.025
S(3,-3)DE		1.582		1.665	0.029
S(4,0)DE		1.579		1.637	0.027
S(0,4)DE		1.564		1.630	0.030
S(4,-4)IDM		0.038		0.031	0.029
S(5,0DE		1.588		1.651	0.048
S(0,5)DE		1.580		1.653	0.035
0°LRE	1.212 ± 0.089		1.274 ± 0.914		0.004
0°SRE	0.955 ± 0.017		0.942 ± 0.019		0.010
0°FIR	0.939 ± 0.023		0.923 ± 0.023		0.005
45°LRE		1.189		1.142	0.010
45°SRE	0.966 ± 0.013		0.956 ± 0.014		0.008
45°FIR		0.946		0.955	0.009

* *Standard deviation*.

**Table 4:** ROC analysis for texture classification using machine learning.

**Method**	**AUC**	**SE**	**95% CI**	**Sensitivity**	**Specificity**	**Accuracy**	***P*-value**
SVM	0.865	0.0419	(0.765, 0.934)	88.90%	73.00%	81.06%	<0.001
LR	0.851	0.0438	(0.749, 0.924)	94.44%	67.57%	81.18%	<0.001
QDA	0.808	0.0509	(0.695, 0.888)	86.10%	62.20%	73.31%	<0.001

*SE, standard error; CI, confidence interval*.

## Discussion

Intratumoral heterogeneity is thought to reflect differences in gene expression, metabolism, angiogenesis and other biological characteristics (29, 30). MRI techniques (including traditional MRI) offer an optimal approach for detecting such heterogeneity in a quick, direct, and non-invasive manner. In recent years, many studies have demonstrated the potential of MRI in the diagnosis of breast cancer (14, 31). Several studies have sought to determine the correlation between breast cancer heterogeneity and histopathological features (32, 33). However, to date, there have not been any studies on investigating the relationship between texture features derived from breast contrast enhanced images and FISH detection assays.

In this study, we used FISH to determine HER2 2+ status and measured the effectiveness of DCE-MRI texture features in classifying this HER2 2+ status. We focused on investigating the consistency between categorization result based on DCE-MRI texture analysis and FISH detection result. We assessed several classical texture features derived from the histogram, co-occurrence matrix, run-length matrix, absolute gradient, autoregressive model and wavelet. No single texture feature was able to classify HER2 2+ status perfectly. Therefore, 30 texture features with statistically significant differences were screened for subsequent classification of HER2 2+ status. It is worth noting that these features were mainly derived from the gray level co-occurrence matrix and the gray-scale run-length matrix, indicating the effectiveness of these two texture analysis methods in HER2 2+ classification. In our study, it must be emphasized that there are two points that can reduce the errors caused by various factors. Firstly, the sample sizes for HER2 2+ positive (37) and negative (36) cases were almost identical, thereby preventing errors due to data imbalances. Secondly, LOOCV was used to avoid classifier over-fitting.

Given the diversity of artificial intelligence methods, future studies adopting advanced methods in machine learning and texture analysis should be conducted to investigate the relationship between texture features and HER2 2+ status. Although patients with HER2 2+ tumors have been well**-**classified, the specific significance of these texture features in pathobiology needs further investigation. In addition, a larger sample size is needed to fully evaluate the robustness of the results from our study. Other relationships between various receptor expression and texture features should be established, and a comprehensive machine learning algorithm should be developed to prospectively predict the expression levels of various kinds of oncogenic protein receptors.

This work presents a preliminary analysis of the use of image characteristics to predict HER2 2+ status. There are several limitations to our study, however. The relatively small sample size in our study limited the statistical analysis. Future studies include increasing sample size to improve upon our current work. In addition, only one radiologist drew the ROI within lesions, and the reproducibility (including inter- and intra-observer differences) was not investigated. Another limitation to our study was that only one slice image (2D) was analyzed, and more adjacent slices (3D level) should be adopted to discriminate HER2 2+ status in the future. We also only focused on the textural features measured using DCE-MRI, and other types of quantitative parameters of DCE-MRI were not incorporated into the analysis, such as *K*_trans_, *K*_ep_, and Karahaliou et al. which may be useful for HER2 2+ characterization (15). We think it would be meaningful to combine DCE-MRI with other imaging modalities, such as diffusion weighted MRI, to further improve prediction accuracy (34). It was only a preliminary research, and the best accuracy was 81% for HER2 2+ status determination based on DCE-MRI features. Hence, the method proposed in this study did not have the ability to replace FISH test, but could be considered as a supplementary tool. With the further development of research in the future, we hope that the accuracy of discriminating HER2 2+ status based on MRI features will be improved.

## Conclusions

This preliminary study using texture analysis to measure HER2 2+ status revealed a highly-promising method with high accuracy. Similar research based on more advanced machine learning algorithms and imaging modalities should be conducted in the future.

## Ethics Statement

The present study was approved by the Ethics Committee of Shengjing Hospital of China Medical University (Shenyang, China). As this was a retrospective study, the requirement for informed consent was waived. All patient information was anonymized.

## Author Contributions

ZJ conducted the experimental progress and manuscript writing. LS conducted the manuscript revision. JY was responsible for the experimental design. HL collected the general data of patients. All authors read and approved the final manuscript.

### Conflict of Interest Statement

The authors declare that the research was conducted in the absence of any commercial or financial relationships that could be construed as a potential conflict of interest.
